# High Strain Rate Deformation of Heat-Treated AA2519 Alloy

**DOI:** 10.3390/ma17235823

**Published:** 2024-11-27

**Authors:** Adewale Olasumboye, Peter Omoniyi, Gbadebo Owolabi

**Affiliations:** Department of Mechanical Engineering, Howard University, Washington, DC 20059, USA

**Keywords:** AA2519, compression, high strain rate, heat treatment, Split Hopkinson pressure bar, stress-strain

## Abstract

This study examined the effects of heat treatment on the microstructure and dynamic deformation characteristics of AA2519 aluminum alloy in T4, T6, and T8 tempers under high strain rates of 1000–4000 s^−1^. A Split Hopkinson pressure bar (SHPB) was utilized to characterize the mechanical response, and microstructural analysis was performed to examine the material’s microstructure. The findings indicated varied deformation across all three temper conditions. The dynamic behavior of each temper is influenced by its strength properties, which are determined by the aging type and the subsequent transformation of strengthening precipitates, along with the initial microstructure. At a strain rate of 1500 s^−1^, AA2519-T6 demonstrated a peak dynamic yield strength of 509 MPa and a flow stress of 667 MPa. These values are comparable to those recorded for AA2519-T8 at a strain rate of 3500 s^−1^. AA2519-T4 exhibited the lowest strength and flow stress characteristics. The T6 temper demonstrated initial stress collapse, dynamic strain aging, and an increased tendency for shear band formation and fracture within the defined strain rate range. The strain rates all showed similar trends in terms of strain hardening rate. The damage evolution of the alloy primarily involved the nucleation, shearing, and cracking of dispersoid particles.

## 1. Introduction

Aluminum and its alloys have generally been classified into the non-heat-treatable (1xxx, 3xxx, 4xxx and 5xxx) and the heat-treatable (2xxx, 6xxx and 7xxx) groups [1,2]. In recent times, there has been need for energy conservation, light weight and high strength, which has resulted in the design of new alloys to meet the demands of various industries [3]. AA2519 is a high-strength aluminum alloy, specifically within the 2000 series, known for its copper content and heat-treatability. Its exceptional resistance to stress corrosion cracking and superior ballistic performance make it a highly desirable material for applications in armored vehicles and aircraft structures [4]. Due to the alloy’s ratio of light weight to high strength, cryogenic properties, high toughness, and good resistance to corrosion cracking, the alloy is used in ballistic applications [5]. Despite its widespread use in ballistic applications, the high-strain-rate response of the AA2519 alloy remains relatively unexplored. The increasing demand for aluminum alloys in aerospace, automotive, and military applications has underscored the need for a comprehensive understanding of their behavior under dynamic loading conditions. This, coupled with the ongoing interest in the plastic deformation of aluminum alloys at strain rates exceeding 10^3^ s^−1^, provides a strong motivation for further research into their failure mechanisms at elevated strain rates [6,7]. The safety and reliability of defense and aerospace structures, particularly in light of the increasing frequency of bird strikes and ballistic impacts, have become paramount concerns [8]. Understanding the material behavior of the AA2519 alloy under high-strain-rate loading is crucial for addressing these challenges. By characterizing the alloy’s dynamic response, researchers can gain valuable insights into its properties and inform the development of improved materials and design strategies for high-performance applications.

Despite the advancements in aluminum alloy applications for structural components, their use in armor systems has been met with varying degrees of success. One significant challenge lies in the limited understanding of their dynamic response across a broad range of strain rates, particularly when faced with modern threats such as improvised explosive devices and explosively formed projectiles [9]. Many of these threats involve dynamic loading conditions, resulting in localized shear deformations. These high-impact strains often lead to the formation of adiabatic shear bands (ASBs), which are narrow regions of intense localized heating and plastic deformation that can serve as sites for material failure [10,11].

Several authors have also studied the dynamic properties of aluminum alloys in different conditions, one of which is Ye et al.’s [12] study on the 2519A alloy processed by interrupted aging. Denser and finer θ′ precipitates were reported to enhance the dynamic yield strength and energy absorption. Similarly, the increased strain rate was attributed to increased grain size and dislocation density [13]. In another study by Pérez-Bergquist et al. [6], the dynamic strain rate test for 5059, 5083, and 7039 aluminum alloys indicated the formation of very compact shear-localized volumes within the sheared zone. The 7039 aluminum alloy consistently exhibited the narrowest shear zones. The shape of the shear bands and the restricted hardening during deformation signified a shift in the mechanism at high strain rates. Higher-resolution orientation image mapping revealed differing levels of crystallographic organization among the shear bands of the three alloys. The mechanical recrystallization of the alloys reportedly influenced high-strain deformation [11].

Several studies have attributed the nucleation of the ASBs to dynamic recovery and recrystallization of the grains [14,15]. Nucleation has also been attributed to inhomogeneities in particle sizes, crystal structure, and geometry [10]. However, works of literature have reported the possibility of microstructure reversal during adiabatic shearing through heat treatments [16]. Therefore, this research aims to study the effects of different heat treatment conditions, such as the T4, T6, and T8, on an AA2519 alloy undergoing high-strain deformation. Although Ye et al. [12] tested A2519 at high strain rates (1300–6900 s^−1^), the present research utilized different strain rates and temper conditions which have not been carried out in any other work of literature. The research will provide sufficient experimental data to make robust predictive modeling and system-level design for the performance of armor systems, as there are limited works of literature on the behavior of heat-treated AA2519 alloys under high-strain deformation.

## 2. Materials and Methods

**Figure 1:**
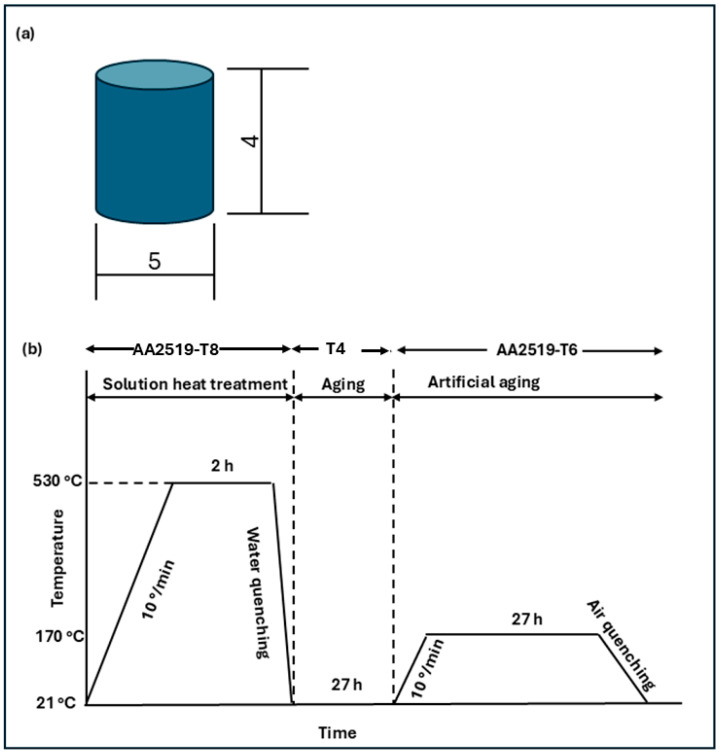
Schematics of (**a**) test sample, (**b**) heat treatment procedure.

The as-received sample is an AA2519 (AlCu6Mn) alloy obtained from NASA under T8 temper conditions of heat treating at 530 °C for 2 h and quenching in water. The samples were cold-rolled with 4% strain (CR4%) to help nucleate fine precipitates. The alloy contained 5.3–6.5% Cu, 0.3% Fe, 0.05–0.4% Mg, 0.25% Si, 0.1–0.5% Mn, 0.05–0.15% V, 0.02–0.1% Ti, 0.1–0.25% Zr, 0.1% Zn and the rest being Al. Cylindrical components measuring 4 mm in diameter and 5 mm in length (aspect ratio, L/D = 0.8) were fabricated from the as-received plates, as shown in Figure 1a. A ratio of 0.8 was adopted to achieve higher strain rates for a uniform uniaxial stress state in the sample.

To investigate the effect of heat treatment on the dynamic response of AA2519, some of the samples were heat-treated to T4 conditions. To obtain the T4 temper condition, six samples were aged at room temperature for 27 h. The T6 condition was achieved by further heat-treating three T4 samples. The samples were heated to 170 °C for 27 h and cooled in air. The schematics of the heat treatment procedure are shown in Figure 1b.

### 2.1. Dynamic Mechanical Response Test of AA2519 Alloy

**Figure 2:**
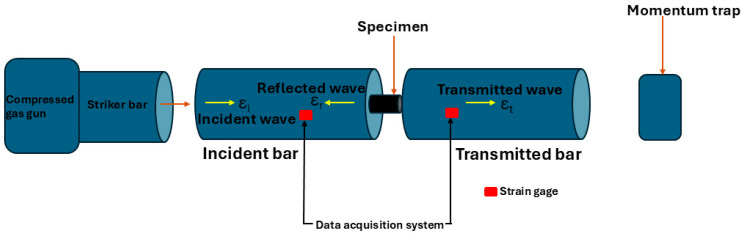
Schematics of the Split Hopkinson pressure bar for compression test.

A high-strain-rate test was carried out on the samples using the Split Hopkinson pressure bar system at room temperature under compressive loading. The impact test was performed by following the procedure of Olasumboye et al. [17,18,19]. The samples were sandwiched between the transmitter and incident bars made of Ti6Al4V alloy (Ө = 13 mm and length of 1.905 m), and 1000 Ω strain gages were mounted 0.947 m from the test specimen on both the incident and transmitted bar to prevent wave interference. The striker bar was fired using a compressed nitrogen gas gun, which set a pulse wave transmitted through the length of the incident bar until it reached the specimen. The pulse oscillated in the specimen until equilibrium was attained, and then the stress or pulse wave continued to the transmitted bar. The data acquisition system then collected strain data from the strain gauges mounted on the incident and transmitted bars. The schematic for the high strain rate test is shown in Figure 2.

This research conducted a compression test on the AA2519-T8, AA2519-T4, and AA2519-T6 samples at a strain rate of 1000–4000 s^−1^ and a step rate of 500 s^−1^. The velocity-time (V-T) data were converted to true stress, strain, and strain rate data using one-dimensional wave theory in Equations (1)–(3) [20,21], where *σ*(*t*) is the true stress; *A_s_* is the cross-sectional area of the specimen; *ε_i_*, *ε_t_*, *ε_r_* are the strains in the incident, transmitted, and reflected bar, respectively; ${\dot{\varepsilon }}_{s}$ is the strain rate; *C*_0_, is the elastic wave velocity of the pressure bar; *A* is the area of the pressure bar; and *E* is Young’s modulus.

(1) $$\sigma \left(t\right)=\frac{AE\left(\varepsilon_{r}+\varepsilon_{i}+\varepsilon_{t}\right)}{2A_{s}}$$

(2) $${\dot{\varepsilon }}_{s}\left(t\right)=\frac{\dot{C_{0}}\left(\varepsilon_{r}-\varepsilon_{i}-\varepsilon_{t}\right)}{l_{s}}$$

(3) $$\varepsilon_{s}\left(t\right)=\int_{0}^{t}\dot{\varepsilon_{s}\left(t\right)dt}$$

### 2.2. Metallographic Preparation and Microstructure Examination of Samples

The initial and post mortem sample surface were prepared by grinding and polishing. The samples were etched using a modified Poulton’s reagent (25 mL methanol, 25 mL HCl, 5 mL HNO3, and a drop of HF). The etching time ranged between 5 and 10 s. The micrographs of the samples pre- and post-deformation were captured using an optical microscope (OLYMPIA BH2-UMA, Feasterville, PA, USA).

## 3. Results and Discussion

### 3.1. Microstructure of the As-Received Pre-Deformed Alloy

**Figure 3:**
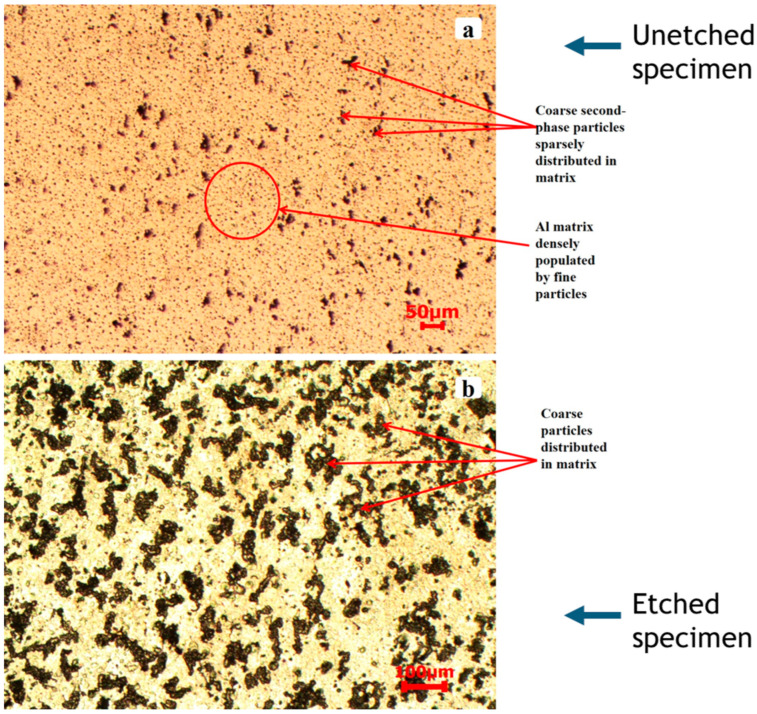
Microstructure of as-received T8 condition sample showing (**a**) coarse and fine secondary particles in the unetched state and (**b**) coarse secondary particles in the etched sample.

The initial microstructure of AA2519-T8 aluminum alloy, as observed in Figure 3a,b, exhibited a coarse particle distribution with sizes ranging from 10 to 123 µm. The average particle size was 36.5 ± 23.2 µm, and the secondary particles displayed a distinctive morphology, similar to observations of Zioko et al. [22] of an average grain size of ~27 µm, with a similar thermomechanical process. Compared to the T4 and T6 tempers, the AA2519-T8 alloy exhibited a higher population density of coarse particles within the observed field of view, despite having a wider grain size distribution. Additionally, Figure 3a reveals a densely populated microstructure containing fine particles without the dendritic structure characteristic of the T6 temper.

In terms of the disconnection of second-phase particles and the uniformity of their dispersion within the matrix, the AA2519-T8 condition surpassed the T6 condition and was comparable to the AA2519-T4 condition. The discrete arrangement of particles; the absence of dendritic structure, as always observed in conventional as-cast aluminum alloys [23]; and the denser population of finely dispersed particles in the AA2519-T8 alloy contributed to enhanced strength and resistance to shear localization during extensive plastic deformation. According to Wang et al. [24], the second-phase particles in AA2519-T8 were intermetallic compounds formed through liquid–solid eutectic reactions during solidification. These phases underwent further transformation during heat treatment and were typically observed to have sizes ranging from one to tens of micrometers [25]. The variation in size of these intermetallic particles can be attributed to factors such as the specific intermetallic phases present in the aluminum alloy, solidification rate, impurity content, and the deformation rate during mechanical and thermomechanical processing [26].

**Figure 4:**
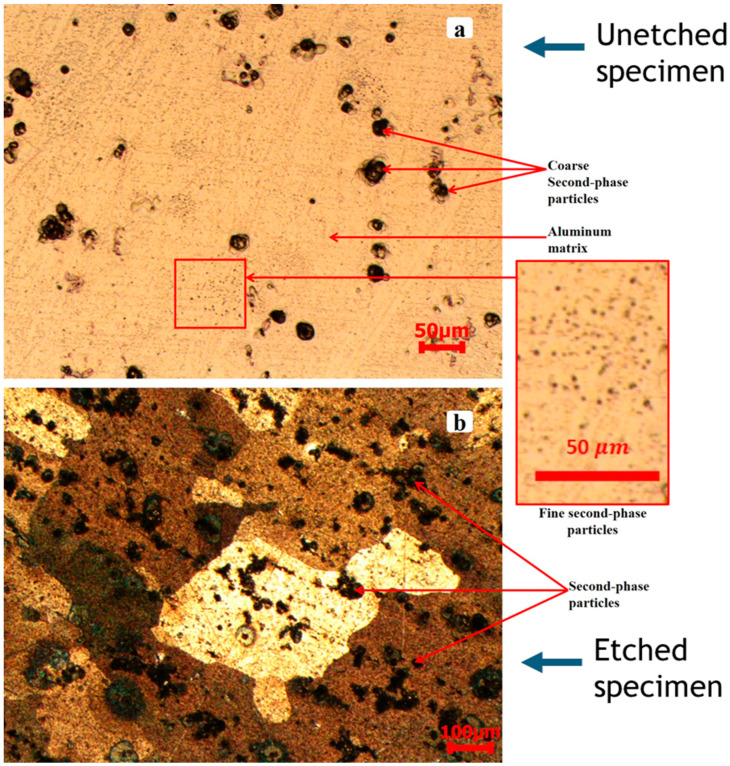
Microstructure of heat-treated AA2519-T4 aluminum alloy prior to deformation: (**a**) without etching, (**b**) with etching.

Figure 4a illustrates the unetched microstructure of AA2519-T4 aluminum alloy. Figure 4b presents the same microstructure post-etching, highlighting the morphology of the second-phase particles. Both micrographs depict dispersed second-phase particles embedded within a continuous α-aluminum matrix, extending throughout the alloy grains [27,28,29]. The second-phase particles are classified into coarse and fine categories according to variations in grain size. The coarse particles exhibited a size distribution from 14 to 84 µm, with an average measurement of 39 ± 15.5 µm, similar to the observations of Jian et al. [30]. The fine particles, which exhibited a round and irregular morphology, were predominantly less than 1 µm in size and were densely distributed throughout the matrix, as illustrated in Figure 4a. Moreover, the fine particles exhibited a higher population density and were more uniformly dispersed than the coarse particles.

**Figure 5:**
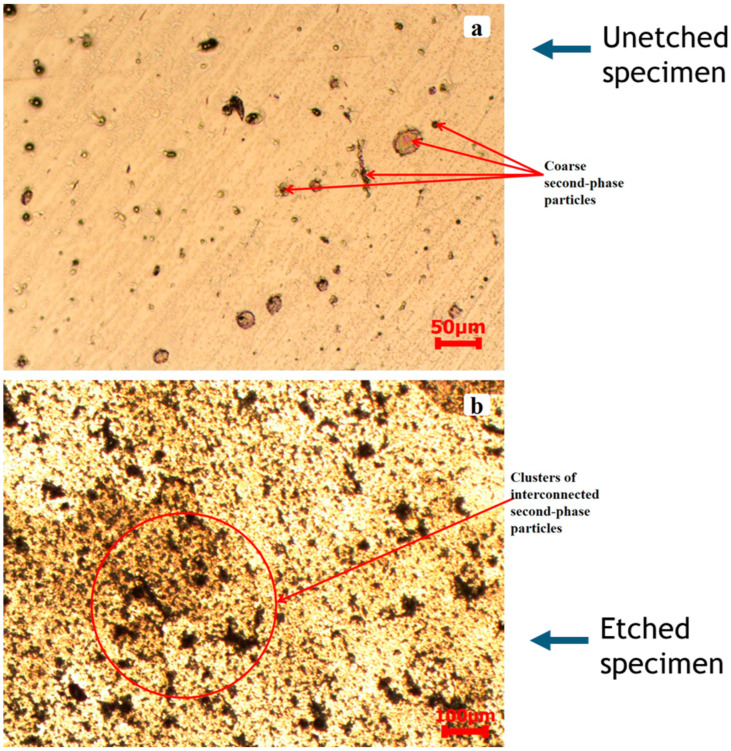
Microstructure of the heat-treated AA2519-T6 aluminum alloy prior to deformation: (**a**) without etching and (**b**) with etching.

Figure 5 presents the pre-deformation microstructure of the AA2519-T6 alloy. Similar to AA2519-T4, second-phase particles can be observed in Figure 5a,b. The coarse particles ranged in size from 7.6 to 61 µm, with an average of 27.6 ± 16.9 µm, while the fine particles were less than 1 µm in size. Unlike the discrete arrangement of coarse particles in AA2519-T4, the coarse particles in AA2519-T6, as shown in Figure 5a,b, exhibited a more interconnected morphology, with some displaying a dendritic structure.

### 3.2. Behavior of AA2519 Aluminum Alloy at High Strain Rates

#### 3.2.1. True Stress–True Strain Behavior

**Figure 6:**
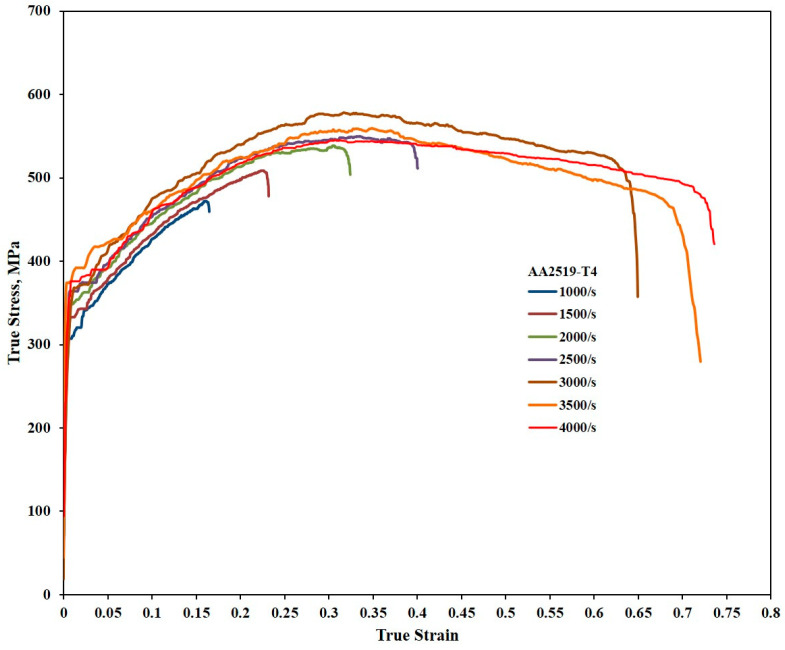
Stress–strain curves of samples with T4 temper condition during compressive loading under high strain rates.

**Figure 7:**
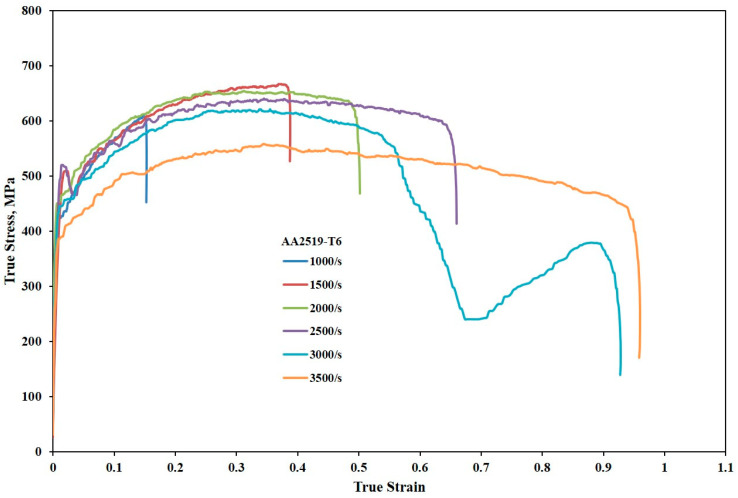
Stress–strain curves of samples with T6 temper condition during compressive loading under high strain rates.

**Figure 8:**
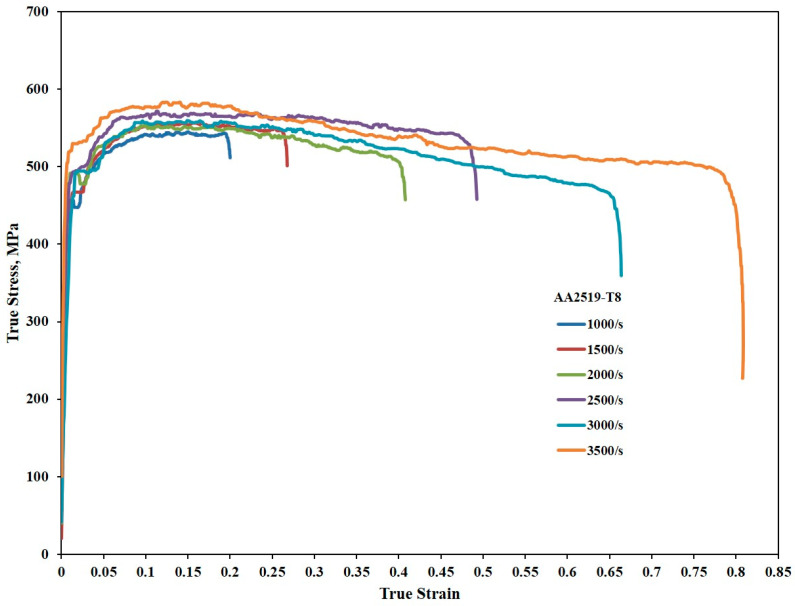
Stress–strain curves of samples with T8 temper condition during compressive loading under high strain rates.

Figure 6, Figure 7 and Figure 8 present the true stress–true strain curves for the AA2519 aluminum alloy in T4, T6, and T8 tempers, obtained at strain rates ranging from 1000 to 4000 s^−1^. True stress and strain are important as they give instantaneous values of stress and strain as the material deforms, i.e., they account for changes in dimension during deformation, unlike the engineering stress and strain, where measurements are based on the original dimensions of the material. The true stress and strain measurements ensure there is an accurate representation of the measurements, thereby reducing overestimation of the material’s strength. All temper conditions exhibited distinct yield points, even at the lowest strain rate of 1000 s^−1^. However, the variation in yield behavior at higher strain rates and the influence of alloy composition highlight the importance of having accurate dynamic yield data for specialized alloys.

After yielding, all curves in Figure 6 exhibited a slight decrease in the rate of stress, which could be attributed to the heat treatment carried out on the samples prior to deformation, significantly increasing the grain sizes of the samples [31]. The plastic deformation region is characterized by strain hardening up to maximum true strains of 0.16, 0.23, 0.30, 0.33, 0.32, 0.35, and 0.31 at strain rates of 1000, 1500, 2000, 2500, 3000, 3500, and 4000 s^−1^, respectively. The total strain attained also varied with the strain rate, reflecting the influence of dislocation multiplication on strain hardening during plastic deformation. The effects of dislocation mobility, density, and arrangement on the strength of metallic alloys have been extensively investigated [32,33,34]. The true stress–true strain curve of the material reached its peak flow stress at a strain rate of 2500 s^−1^, with the peak occurring at varying true strains. After reaching the peak, strain hardening slowed, leading to a slight but steady decrease in flow stress over various strains depending on the strain rate. This phenomenon, known as thermomechanical instability, is attributed to the conversion of approximately 90% of plastic work into heat. A sharp drop in flow stress was observed at a higher strain rate of 3500 s^−1^. While strain hardening is generally associated with dislocation accumulation, stress collapse has been linked to thermal softening caused by the excessive heat generated during deformation [35,36,37].

Additional mechanisms that contribute to strain localization in aluminum alloys under impact loading include dynamic recrystallization and the nucleation, growth, and coalescence of voids [38,39,40]. The rapid decrease in flow stress during flow softening is attributed to thermomechanical instability arising from intense adiabatic heating within localized regions of the specimens at high strains. The interplay between strain hardening and thermal softening during plastic deformation ultimately determines the maximum peak flow stress and the overall deformation profile [41].

Despite achieving a total strain of approximately 0.74 at a strain rate of 4000 s^−1^, the AA2519-T4 alloy exhibited a relatively minor degree of flow softening, as illustrated in Figure 6. Macroscopic examination of the tested specimen revealed no fractures, and the limitation imposed by specimen size prevented further testing at strain rates exceeding 4000 s^−1^. Consequently, the total strain to failure was anticipated to be greater than 0.74, indicating the alloy’s inherent high ductility, a characteristic typical of AA2519 aluminum alloys.

This property behavior was found in a study by Zuiko et al. [22] on AA2519 to be significant at high temperatures ranging from 450 to 535 °C. In the study, Zuiko et al. determined the highest elongation-to-failure of ~750% at a temperature of 525 °C. The AA2519 alloy demonstrated substantial strain hardening across various strain rates, resulting in excellent strength characteristics. It has been reported that AA2519 exhibits a yield strength approximately 20% higher than AA2219, another Al-Cu alloy, when subjected to similar processing conditions [13,42]. However, it was reported that the strain hardening rate decreased with an increasing strain rate.

The AA2519-T6 alloy exhibited distinct yield points, as shown in Figure 7, but with a steeper initial decline in stress compared to AA2519-T4 within the strain rate range of 1500 to 2500 s^−1^. During this initial stress decline, deformation remained heterogeneous. The true strains corresponding to the peak flow stress, marking the end of homogeneous deformation, were 0.15, 0.37, 0.31, 0.35, 0.34, and 0.34 at 1000, 1500, 2000, 2500, 3000, and 3500 s^−1^, respectively. Unlike AA2519-T4, AA2519-T6 displayed a two-stage strain hardening and flow softening behavior after yielding, with a more pronounced initial strain hardening stage and a less significant second stage. The stress–strain curve reached its second peak prior to the domineering effect of thermal softening. This trend was reported in the AA2219 aluminum alloy [43] when deformed at a strain rate of about 5000 s^−1^. At a strain rate of 3500 s^−1^, the AA2519-T6 alloy exhibited minimal strain hardening compared to lower strain rates. Additionally, the peak flow stress was lower than at lower strain rates. This decrease in work hardening and subsequent drop in flow stress with continued plastic deformation might be attributed to reduced material ductility at high strain rates. Macroscopic examination of the tested specimen revealed a complete fracture along a circular path near the compressed surface. The total strain to failure was 0.96.

In AA2519-T8, as shown in Figure 8, the true strains recorded at peak flow stress leading to the end of homogeneous deformation were 0.15, 0.16, 0.10, 0.11, 0.15, and 0.12 at strain rates of 1000, 1500, 2000, 2500, 3000, and 3500 s^−1^, respectively. The maximum true strain attained by AA2519-T8 (0.16) was lower than that of AA2519-T4 (0.35) and AA2519-T6 (0.37). Flow softening began at strains less than 0.2 for all strain rates, but the rate of flow softening was relatively minor, as observed in the true stress–true strain curves.

#### 3.2.2. Strain Hardening Behavior

The strain hardening behavior of the samples was thoroughly investigated by analyzing the dynamic stress–strain curves presented in Figure 6, Figure 7 and Figure 8. Using the homogeneous portions of these curves, strain hardening exponents were determined for the T4, T6, and T8 tempers at various strain rates. These exponents were then employed to construct plastic flow stress–true strain curves based on Hollomon’s power law, providing valuable insights into the strain hardening response of the materials [44]. The strain hardening exponents were further utilized to construct strain hardening rate (SHR) curves, allowing for an in-depth analysis of how the material’s work hardening behavior evolved with increasing strain and strain rate across all the temper conditions.

#### 3.2.3. Strain Hardening Exponents

**Figure 9:**
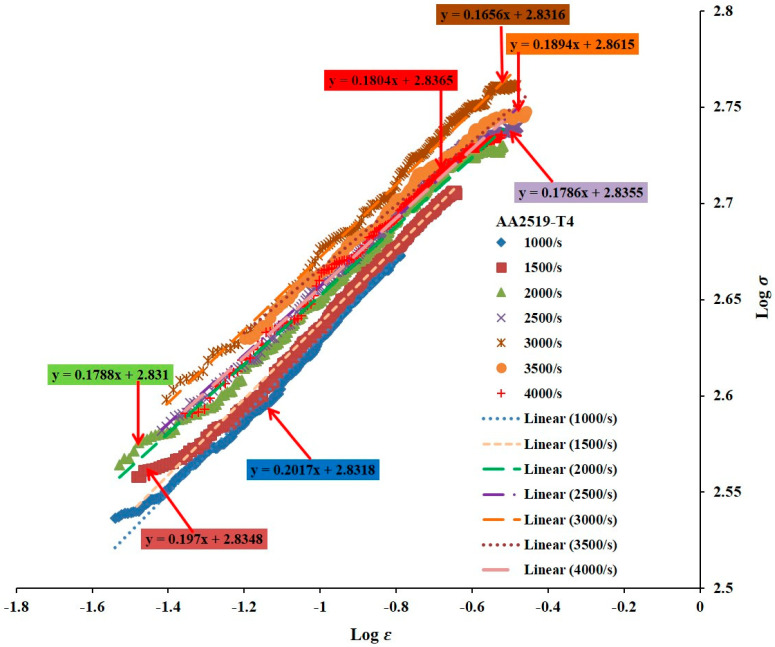
Logarithmic curves of true stress–strain of T4 condition sample within the homogenous deformation region.

**Figure 10:**
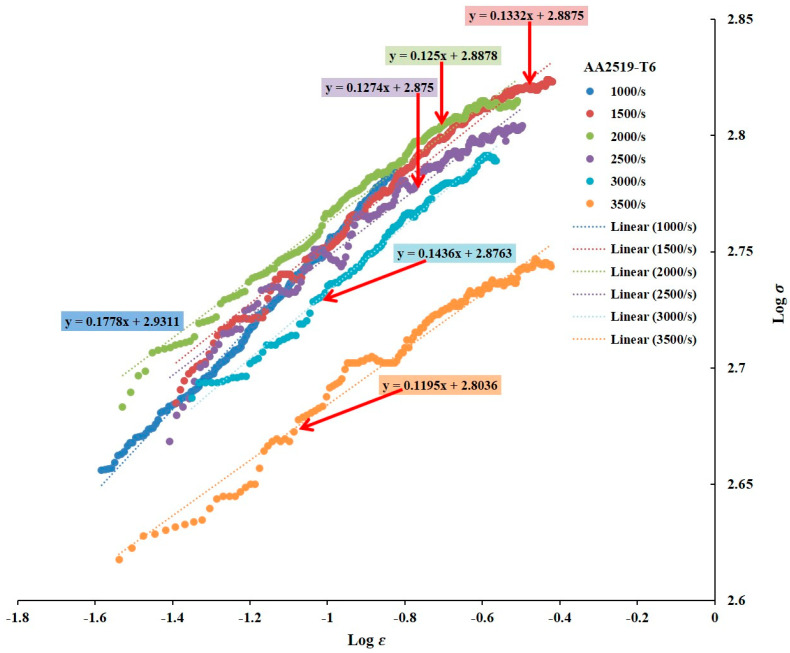
Logarithmic curves of true stress–strain of T6 condition sample within the homogenous deformation region.

**Figure 11:**
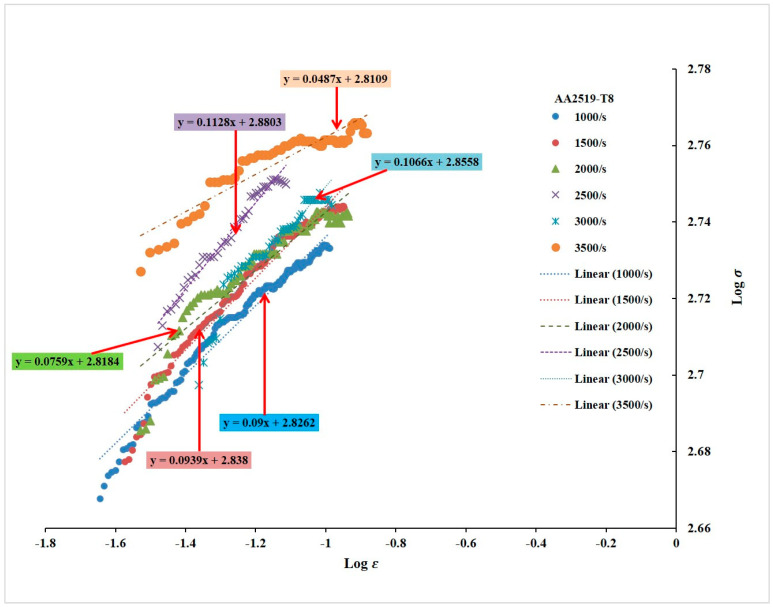
Logarithmic curves of true stress–strain of T8 condition sample within the homogenous deformation region.

Figure 9, Figure 10 and Figure 11 depict the strain hardening exponents of AA2519 aluminum alloy in T4, T6, and T8 tempers at various strain rates, respectively. A clear trend emerged, with the linearity of the logarithmic stress–strain data improving from T4 to T8. The strain hardening exponent decreased at each strain rate, ranging from 1000 to 3500 s^−1^. This could be attributed to the fact that AA2519-T8 contained an initial amount of work hardening [22] before being subjected to compressive loading. As expected, AA2519-T8 exhibited lower strain hardening behavior than AA2519-T4 and AA2519-T6, resulting in the lowest strain hardening exponents at all tested strain rates. These findings validate the use of the strain hardening exponent as a reliable indicator of strengthening during plastic deformation. The increased strain hardening exponents with increasing strain rate at each material’s tested range further confirm this correlation. Moreover, the limited strain-hardening capacity of AA2519-T8, as evident in Figure 8, is reflected in its lower maximum true strain of 0.16 compared to the other tempers.

#### 3.2.4. Plastic Flow Stress–Strain Curves

**Figure 12:**
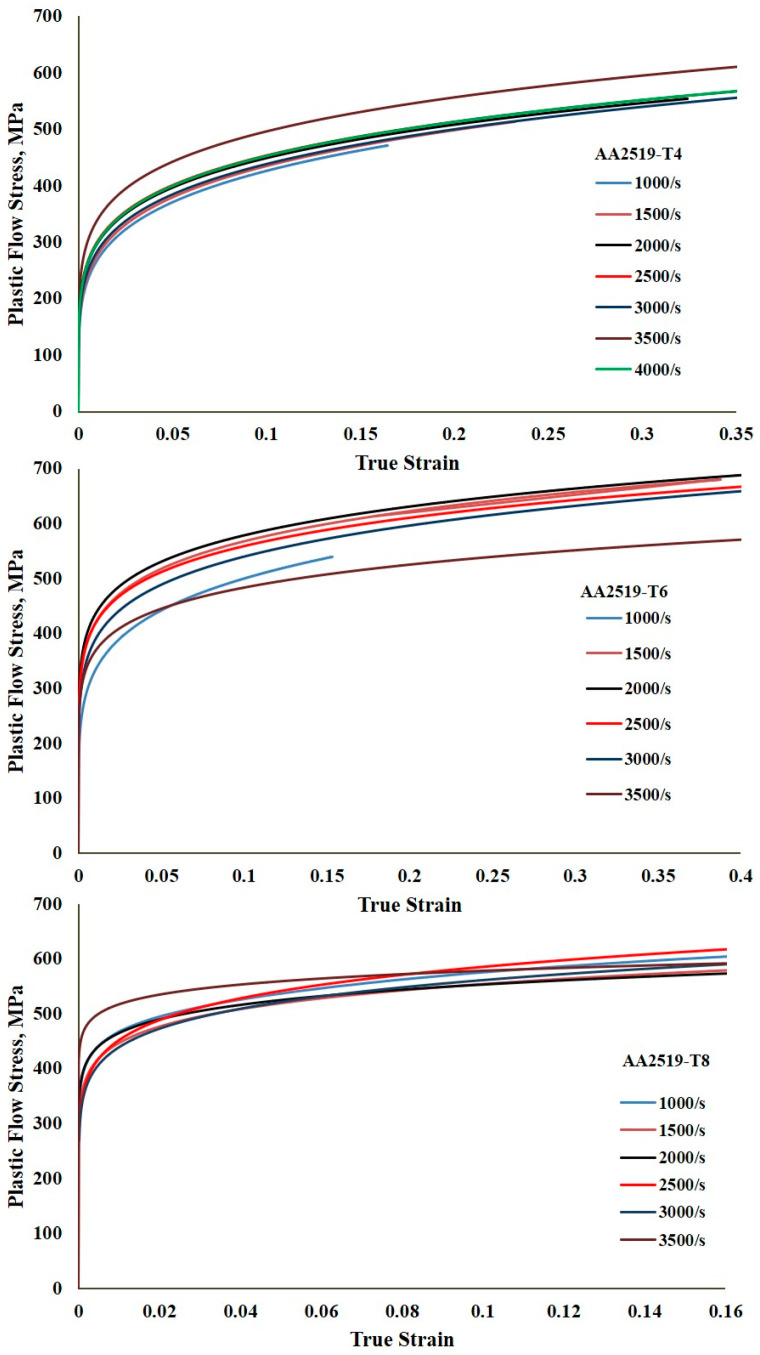
Strain hardening responses of samples at different temper conditions.

Power law relationships were established for each material based on the n and K values derived from previous analyses (Figure 8, Figure 9, Figure 10 and Figure 11). A noteworthy observation is that AA2519-T6 consistently exhibited higher K values across the tested strain rates, except for 3500 s^−1^, where fracture occurred. This enhanced strength in AA2519-T6 can be attributed to the synergistic effects of high strain hardening, elevated strain hardening rate, and the precipitation of strengthening phases, characteristic of its aging treatment [45,46]. The experimental true strains are collapsed into Figure 12, presenting the plastic flow stress–true strain curves. These curves provide a clearer visualization of the material’s response during homogeneous deformation at varying strain rates. Notably, the curves for AA2519-T4 exhibit a steeper slope than those of AA2519-T6 and AA2519-T8. This reduced slope suggests a lower strain hardening exponent.

#### 3.2.5. Strain Hardening Rate Response

**Figure 13:**
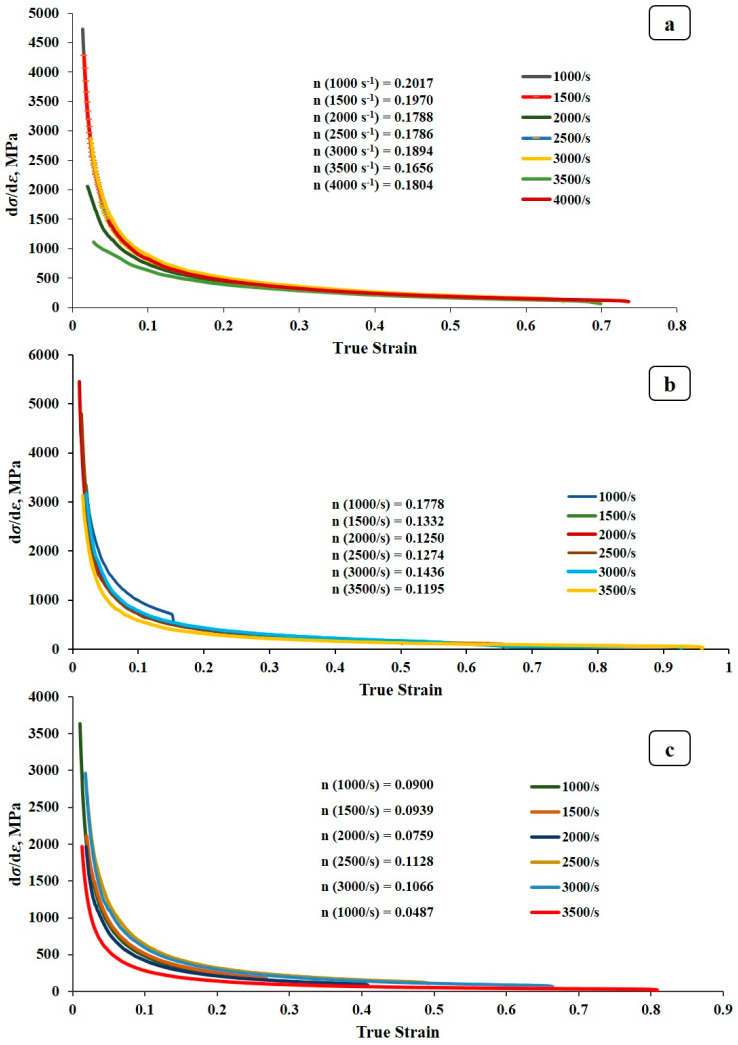
Strain hardening rate and true plastic strain curves of (**a**) AA2519-T4, (**b**) AA2519-T6, and (**c**) AA2519-T8.

**Figure 14:**
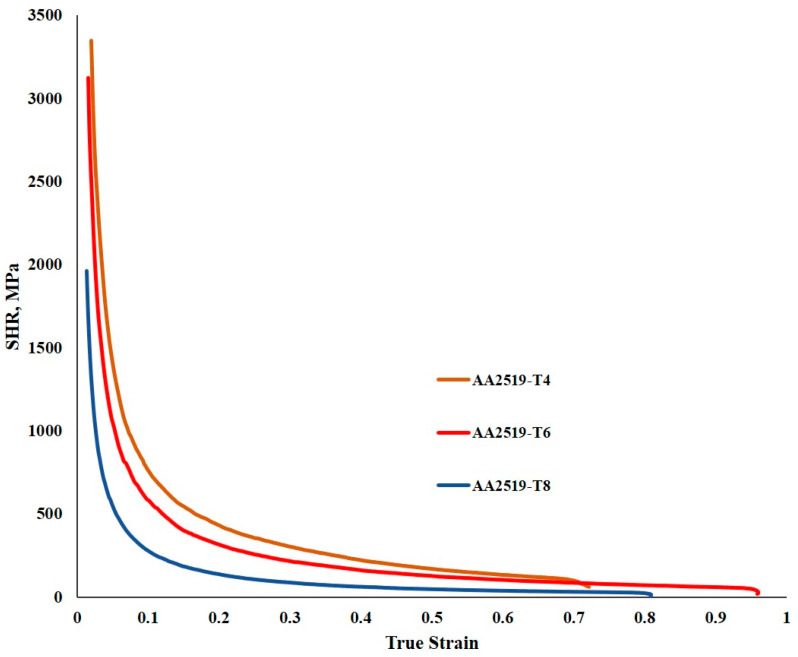
Strain hardening rate and plastic strain of samples in the temper conditions at 3500 s^−1^.

Figure 13 illustrates the shear hardening rate (SHR) curves for the materials subjected to high strain rates. All three materials demonstrated notable initial strain hardening rates, which decreased swiftly as the strain increased. AA2519-T6 exhibited the highest initial SHR, measuring around 5500 MPa, while AA2519-T4 and AA2519-T8 showed values of 4500 MPa and 3500 MPa, respectively. The reduced initial SHR of AA2519-T8 can be linked to its previous work history. The SHR values gradually declined, ranging from 1000 to 3500 s^−1^ across all samples. The influence of the strain rate on SHR curves was relatively minor; however, the impact of the temper condition was more pronounced, as indicated by the significant increase in SHR from T8 to T4 in Figure 14.

### 3.3. Investigation of Microstructure Changes During Deformation of AA2519 Aluminum Alloy in T4, T6, and T8 Temper Conditions

**Figure 15:**
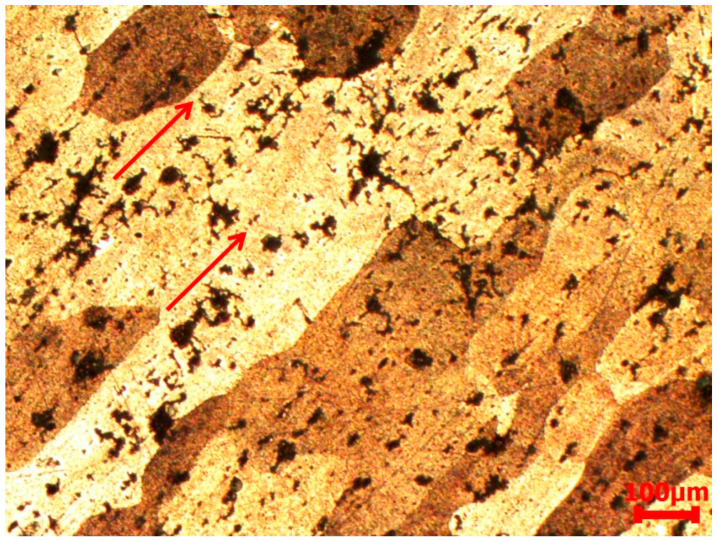
Microstructure of the deformed T4 condition sample at 3500 s^−1^.

Figure 15 presents the microstructure of AA2519-T4 alloy deformed at 3500 s^−1^. In contrast to its initial state, which featured a relatively discrete distribution of coarse and fine second-phase particles, the deformed microstructure exhibited a more interconnected arrangement of these particles, aligned along the shear-flow direction (indicated by red arrows). The previously round, irregular, and coarse secondary particles evolved into dendritic structures following extensive plastic deformation. Similar observations were also reported by Yang et al. [29]. The distribution of deformed and elongated intermetallic particles was observed within and across grains and grain boundaries, respectively.

**Figure 16:**
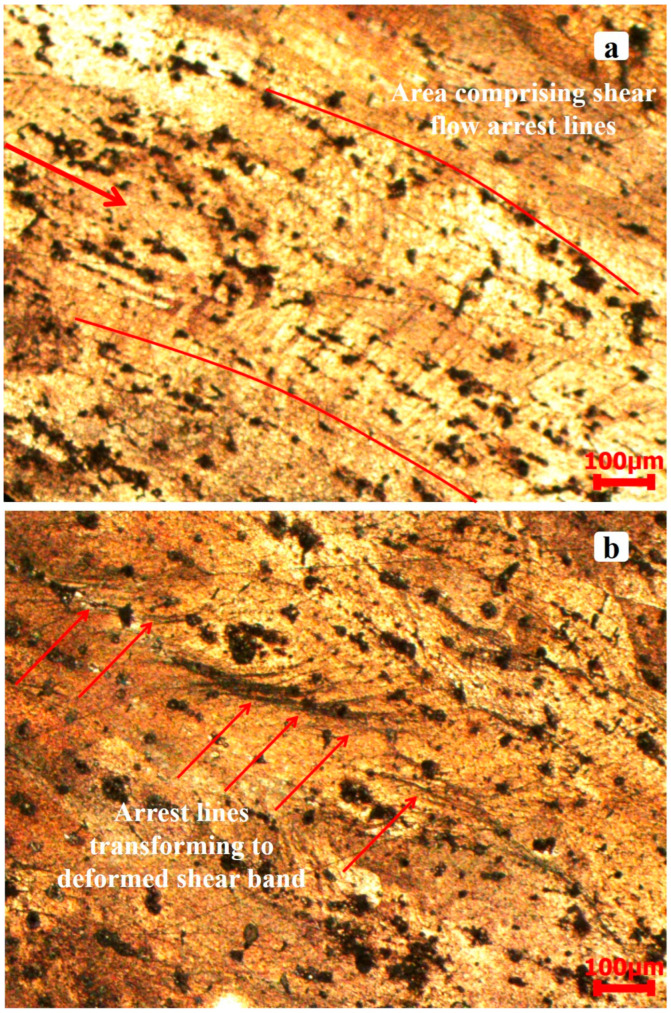
Microstructure of the deformed T4 condition sample from different locations on the specimen at 4000 s^−1^, showing (**a**) shear flow arrest lines and (**b**) deformed shear bands.

**Figure 17:**
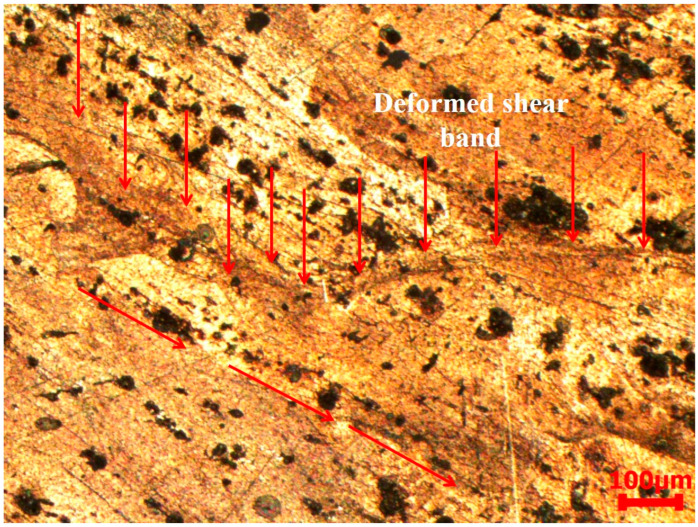
Microstructure of the deformed T4 condition sample showing shear flow arrest lines transforming to the deformed shear band at 4000 s^−1^.

Figure 16 presents the deformed microstructure of the alloy at 4000 s^−1^. Intermetallic particles exhibited further elongation at this strain rate and arranged into a band-like pattern (Figure 16a). A distinctive feature observed within these bands was the presence of “arrest lines” opposing the material’s flow direction. Figure 16b illustrates a comparable characteristic at a different position along the shear-flow direction, where the arc-like lines seem to have been separated due to shearing. The area between the divided arcs shows a significant decrease in coarse particle size, indicating possible particle dissolution. These split arcs are hypothesized to have transformed into deformed shear bands, as illustrated in Figure 17. The heterogeneous deformation observed in AA2519-T4 at 4000 s^−1^ can be attributed to intermetallic particle elongation, shearing, and cracking. The cracking of Fe-rich intermetallic particles has been reported in the literature on AA6061 aluminum alloy. Agarwal et al. [47] examined the alloy’s susceptibility to Fe-rich particle formation and the influence of loading direction on the fraction of damaged particles. Particle cracking was observed regardless of loading direction, whether it was perpendicular or parallel to the extrusion axis. However, the number fraction of damaged particles increased in the perpendicular direction, while it decreased and eventually reached saturation in the parallel direction [48].

**Figure 18:**
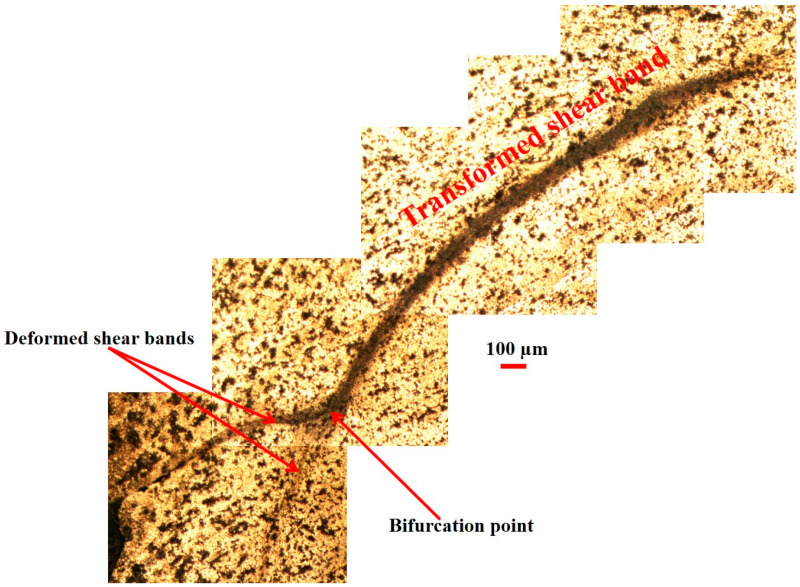
Microstructure of T6 condition sample deformed at 3000 s^−1^, showing the presence of the two ASB types and bifurcation of the transformed band.

**Figure 19:**
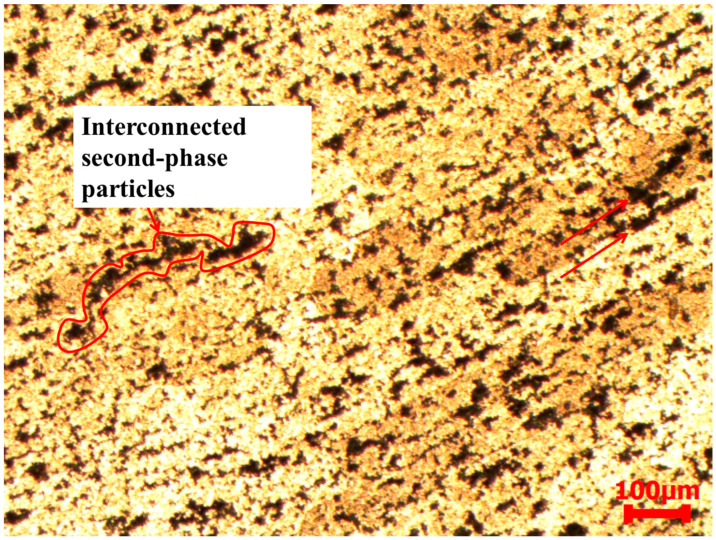
Microstructure of T6 condition sample captured outside ASB from the deformed specimen at 3000 s^−1^.

**Figure 20:**
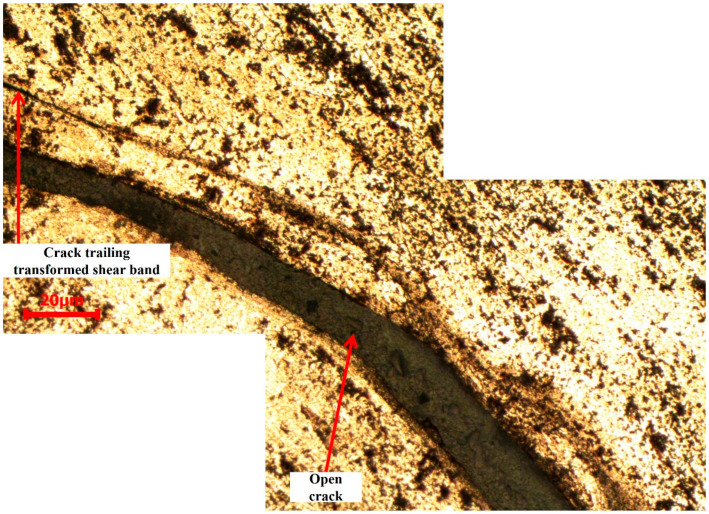
Microstructure of T6 condition sample deformed at 3500 s^−1^, revealing an open crack and ASB.

While no evidence of shear bands was observed in AA2519-T6 aluminum alloy at strain rates below 3000 s^−1^, deformed and transformed bands were identified at 3000 s^−1^. Figure 18 presents the microstructure of shear bands formed in AA2519-T6. The initial, narrower band was observed to bifurcate and branch into two other bands, extending into the alloy matrix. As indicated in the figure, the bifurcated branches exhibited more pronounced deformation than transformation. This is attributed to the insufficient driving force at the tip of the transformed band to produce large strains, a prerequisite for transformed band formation. The dark brown color of the transformed band suggests partial dissolution of intermetallic particles along the shear band. Notably, the coarse second-phase particles outside the shear bands remained relatively unchanged, further supporting the heterogeneous nature of deformation [49]. As depicted in Figure 19, coarse second-phase particles were observed outside the well-defined shear band to align and connect sequentially. These interconnected and elongated particles clustered in the direction of material flow, forming a serration pattern on the specimen surface. This phenomenon may be attributed to dynamic strain aging. In AA2519-T6, deformed at 3500 s^−1^, an open crack was observed to have formed adjacent to a fully developed transformed band (Figure 20). This implies that the newly changed band may have originated from the source of the initial crack. The literature has documented mechanisms for crack formation in altered bands, including microvoid nucleation and propagation within these bands. Microvoids may initiate at constituent particles or inclusions when the applied stress is beyond the critical threshold for particle fracture or particle–matrix decohesion [36,50].

The formation of microvoids was observed to be promoted by the decohesion occurring between the smaller second-phase particles and the alloy matrix [38]. Microvoids are prone to nucleation, growth, and coalescence within shear bands due to the intense localized heat and shear stress. While microvoids may be present, they might not be readily identifiable in micrographs obtained through optical microscopy (OM) due to etching reactions with second-phase particles that obscure the contrast between microvoids and the surrounding matrix. The strain within the transformed band is significantly higher than that in the deformed band, making crack initiation more likely to occur at the root of the transformed band.

**Figure 21:**
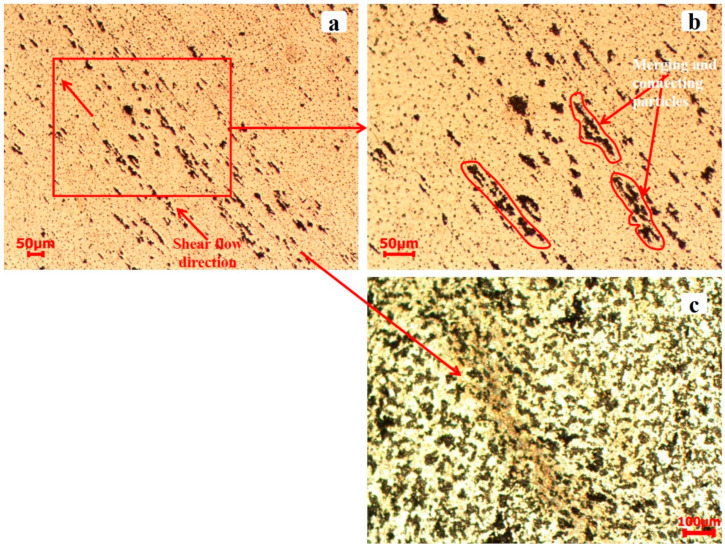
Microstructure of T8 condition sample deformed at 3500 s^−1^: (**a**) secondary particles agglomerating along the shear flow direction, (**b**) particles merging and linking, (**c**) evolution of the deformed shear band.

Figure 21 presents the micrograph of AA2519-T8 aluminum alloy deformed at 3500 s^−1^ in both unetched (a and b) and etched (c) conditions. Similar to the damage evolution observed in the T6 temper, elongated and interconnected coarse second-phase particles aligned along the shear-flow direction, exhibiting signs of strain-induced deformation. As shown in Figure 21c, etching revealed an evolving band within which particles had partially dissolved, with no visible cracks on the metallographic plane.

## 4. Discussion

In this research, the dynamic response of AA2519 in three temper conditions, T4, T6, and T8, was examined at a high strain rate between 1000 and 4000 s^−1^. The Split Hopkinson pressure bar was used to carry out the test. Before deformation, the microstructure shows dispersed second phase particles with α-aluminum across the grains in the T4 tempered samples. The grain sizes ranged from 14 to 84 µm, and more fine grains were present. The T6 condition had particle sizes ranging from 7.6 to 61 µm, which were finer than those of the T4 temper condition as a result of the fine precipitates in the microstructure, which promoted the mechanical properties of the sample. The T8 condition had grain sizes ranging from 10 to 123 µm, and the grains appear coarser than those of the T4 and T6 temper conditions.

The true stress–strain response accounts for the mechanical behavior of the material under high strain. It provides information on the flow stress and strain hardening behavior of the material. The maximum dynamic yield strength of the T4 sample (391.96 MPa) was recorded at a strain rate of 3500 s^−1^, while that of the T6 had a maximum yield strength of 519 MPa at 2500s^−1^ and T8 had a maximum yield strength of 520.86 MPa at 3500 s^−1^, signifying a 24.7% increase in strength over the T4 condition. The insignificant difference between the yield strength of the T6 and T8 could be attributed to the similar aging processes of the two conditions. The T4 plastic region was characterized by strain up to a maximum of 0.35 at 3500 s^−1^. While there was a steeper decline in stress in T6 between 1500 and 2500 s^−1^ when compared to T4, the maximum strain was recorded as 0.37 at 1500 s^−1^. The T8 sample recorded a much lower maximum strain of 0.16 at 1500 s^−1^.

The strain hardening coefficient *n* is known to increase as strength reduces [51], with the T4 condition having a maximum *n* value of 0.2017 at 1000 s^−1^, followed by 0.1778 at 1000 s^−1^ for the T6 condition and 0.1128 at 2500 s^−1^ for the T8 condition, signifying an improved ability to resist deformation.

The microstructure after deformation was characterized by larger grain sizes, and the grains aligned along the shear flow direction. At a high strain rate, arc-like arrest lines appeared, opposing the material flow within the evolving band and direction. The arc-like lines were observed to have transformed into shear bands, which were less coarse than the surrounding microstructure, implying that the material underwent heterogeneous deformation at high strain rate of 4000 s^−1^. However, in samples below 3000 s^−1^, there was no observation of shear bands for any temper samples.

The T6 and T8 temper conditions were not tested at strain rates greater than or equal to 4000 s^−1^, as a result of the formation of adiabatic shear bands at 3500 s^−1^. It is recommended for further studies to examine the creep and mechanical behaviors of the alloy under combined high-temperature and high-strain-rate environments. In addition, advances in manufacturing such as additive manufacturing (AM) make it imperative to examine how additive manufacturing methods (e.g., selective laser melting or electron beam melting) affect the mechanical properties of the alloy at high strain rates.

## 5. Conclusions

A comprehensive characterization of AA2519 aluminum alloy in T4, T6, and T8 tempers was conducted at high strain rates (1000–4000 s^−1^) using compression testing. The study encompassed both macroscopic and microstructural analyses to elucidate the material’s high-strain-rate behavior and provide experimental data for modeling its constitutive response at elevated strain rates.

1. The results indicated varied deformation across all three temper conditions. The mechanical behavior of each temper is influenced by the strength properties of the material, which are determined by the type of aging and the subsequent transformation of strengthening precipitates along with the initial microstructure. The damage evolution modes observed across all three conditions were consistent, characterized by the nucleation, coalescence, and shearing of dispersoid particles, along with the propagation of ASB.
2. The maximum dynamic yield strengths for AA2519-T4, T6, and T8 were measured at 392 MPa, 509 MPa, and 520 MPa, respectively. The peak flow stresses measured were 578 MPa, 667 MPa, and 583 MPa, respectively. AA2519-T6 demonstrated its maximum dynamic yield strength and flow stress at a rate of 1500 s^−1^, similar to the values recorded for AA2519-T8 at 3500 s^−1^. AA2519-T6 exhibited negative strain rate sensitivity, which suggests dynamic shear aging, and was determined to be the most prone to adiabatic shear band formation. In AA2519-T6, a transformed band that resulted in crack nucleation and propagation was observed at a strain rate of 3000 s^−1^.
3. AA2519-T4 demonstrated the lowest dynamic yield strength and peak flow stress compared to the other two tempers. Although it exhibited reduced strength, it showed the lowest tendency for shear band formation. At a strain rate of 4000 s^−1^, although ASBs were observed to form, AA2519-T4 demonstrated a degree of resistance by developing arc-like “arrest lines” that seemed to hinder shear localization. The shearing of the “arrest lines” resulted in the formation of deformed bands in AA2519-T4 at a strain rate of 4000 s^−1^. In comparison to AA2519-T4 and T6, AA2519-T8 demonstrated significant benefits, such as a more uniform distribution of both coarse and fine second-phase particles within the aluminum matrix, as well as enhanced control over the nucleation of dispersoid particles from impurity atoms during deformation.

## Data Availability

The data that support the findings of this study are available from the author upon request.
